# 

**Published:** 1876-03

**Authors:** 


Transactions of Mississippi State Medical Association. Pp. 32.

President, M. S. Croft, M.D.; Vice-Presidents, B. A. Vaughn, M.D., Columbus; E. W. Hughes, M.D., Grenada; W. H. Armstead, M.D., Vaiden; John Brownrig, M.D., Columbus; Permanent Secretary, R. A. New, M.D., Port Gibson; Corresponding Secretary, J. W. King, M.D., Vicksburg; Treasurer, J. A. Campbell, M.D., Shuqualak; Orator, E. G. Banks, M.D., Clinton.

---

Alcohol: Six Lectures. By Benj. W. Richardson, M.A., M.D., F.R.S. Pamphlet; pp. 190.

---

Series American Clinical Lectures, edited by E. C. Sequin, M.D., on Physiological Antagonism as applied to treatment of Febrile State. By Roberts Bartholomew, M.A., M.D. G. P. Putnam's Sons. Pamphlet; pp. 22.

---

Transactions Colorado Medical Society, Third and Fourth Annual Sessions. H. A. Lemen, Secretary, Denver, Colorado. Pamphlet; pp. 76.

---

Medico-Surgical Evidence in New-Born Child. By J. B. Gaston, M.D., Montgomery, Ala. Pamphlet; pp. 6.

---

Treatment of Scrofulides (Lupus.) By H. G. Piffard, A.M., M.D. Pamphlet; pp. 11.

---

Aphonia: Its Causes and Treatment. By Wm. Porter, M.D. Pamphlet; pp. 16.

### INDEX TO VOLUME XIII.

Abortion, law of ............... 370
Abscess, mammary..... 76, 360, 646
Abstract ..................... 762
Acid, salicylic ................ 161
Carbolic, as a disinfectant... 161
Carbolic, as a local anæsthetic 219
Address, Valdic ................ 1
Advertising .................... 377
Alexander, L. ............. 79, 466
A Lost Art ..................... 298
Alcohol, its action ............ 402
Elimination of by respiration 426
Lectures on .................... 759
American Public Health Ass'n... 507
American Clinical Lectures, series of ......................... 859
American Medical Association... 62
Proceedings ............ 171, 189
Ammenorrhœa, case of ........... 231
Ammonia, carbazotate of......... 161
Amputation, methods of.......... 359
Anatomist, ode to his mistress... 39
Anatomical rooms, ventilation of. 441
Anæsthesia, in obstetrics....... 225
Anus, absence of................ 171
Anthelmintics................... 499
Aphonia, causes and treatment... 759
Argumentum ad hominem.......... 295
Army medical staff.............. 64
Asthmatic mixture .............. 361
Aspirating the bladder, simple method of ..................... 414
Atlanta Medical College...... 1, 760
Atalanta Academy of Medicine,
29, 94, 151 221, 270, 474, 551, 603
655, 731
Bacteria and Septicæmia......... 789
Barry, Daniel................... 340
Battey, Robert.................. 257
His operations ........ 481, 713
Belladonna, value of in scarlatina, 628
Bibliography, 62, 187, 247, 311, 375
439, 505, 571, 631, 698
Bloodletting...... ...... 391, 603
Blood, coloring matter of....... 412
Blood-letting .................. 752
Board of Health, organization of, 255
Bolling, Dr., Address........... 187
Bone, diseases of............... 29
Boils, sulphuric acid in,....... 630
Brain, physiology of............ 424
Brandon, D. S................... 193
British Medical Association, abstract of ..................... 427
Bronchitis, chronic. ...... 61
Brown sugar, syrup of, antiseptic 58
Bruce, W. N. ................... 539
Bulkely, L. Duncan.............. 679
Burking, crime of............... 382
Calhoun, A. W.,
33, 146, 395, 471, 587, 670, 740
Calculi, biliary............. .... 432
Campbell, H. F.................. 129
Cancer Doctors.................. 239
Cancer ......................... 42
Operation for.................. 603
Cantharides, dangers of......... 367
Cataract, in the aged .......... 98
Catarrh, dust a cause........... 558
Catching Cold .................. 565
Cathartic, agreeable ........... 690
Cauliflower Excrescence ........ 50
Cæcum, impaction of............. 661
Cervix Uteri, bilateral section of. 413
Cephalalgia, nitrite of amyl in .. 627
Cheap Office Sign............. .. 366
Cherry-Stones, removal of, from rectum.......................... 746
Child killed by Chicken......... 319
Chills and Fever, treatment of... 393
Chloroform, milk kept by........ 690
In obstetric practice.......... 340
Chloroform Asphyxia, how to prevent.......................... 755
Chloral Hydrate................. 367
Use of......................... 494
External use of ............... 374
Intravenous injection of ...... 750
Cinchona Alkaloids, cheaper .... 356
Cincho-Quinine ............ 46, 64
Clinic Surgical, Atlanta Med. Col.
596, 673
Eye and Ear, 33, 395, 471, 600, 670
Cod Liver Oil and Phosphorus, formula for ...................... 630
College of Physicians, Philadelphia, trans. of.................. 631
Convulsions, puerperal.......... 604
Copeland, A. B.................. 269
Cornea, Fistula of.............. 168
Correction...................... 448
Cotton Worm, habits of.......... 60
Cromwell, B. W.................. 641
Couvert, A. ............... 150, 153
Croup and Diphtheria............ 497
Croup, membraneous...... 432, 496
Operation for ................. 562
Condom, fifteen uses for........ 241

Cyst, peritoneal................ 228
  Ovarian, multiple .......... 164
  Time for operating......... 165
Damiana ..................... 291
Deafness and Blindness, due to tapeworm .................. 431
Death, delivery after............ 59
Demlit Dispensary, priv. class of 679
Denidation..................... 358
Dekle, T. S. .................... 452
Diagnosis, errors of............. 150
Diet, Chambers' Manual of...... 313
Diphtheria........ ............ 40
  Communicability of........ 744
  Treatment of.......... 466, 6[illegible]3
Displacements, uterine ......... 449
Doctors ....... .............. 237
Doses, infinitessimal............ 126
Dropsy, cardiac, digitalis in..... 56
Drunkards, treatment of ........ 364
Dry Cupping ................... 531
Duncan, D. P.................... 393
Dugas, L. A.......278. 460, 526, 646
Dysentery, treatment of......... 247
  Natural history of ......... 307
Dysmenorrhœa, membraneous, 274, 257, 400
Dysmenorrhœal Membrane. 221, 273
Ear, larvæ in................... 619
Eczema........................ 424
  Tinct. arnica a cause of..... 629
Editorial, 62, 127. 189, 249, 314, 377 441, 508, 572, 634, 700
Electricity ..... ............. 154
Epilepsy, cure of............... 98
Epistaxis, quinine in........... 61
  Device for................. 508
Erysipelas.... ............... 276
Ether vs. chloroform........... 223
Ex-Confederate Army and Navy Officers, meeting of .......... 448
Eye Ball, dislocation of......... 587
  Extirpation of.............. 660
Eye Lid, wound of.............. 464
Fever, Intermittent, treatment of 452
  Typhoid ..... ............ 57
  Observations on............ 321
  Cause of.................. 565
  Puerperal..... ........... 76
Fees in New York City.......... 368
  Extra.. .................. 570
Fistula, anal...... ........... 97
  In consumption............ 97
  In cornea.................. 168
Florida, climate of.............. 492
Fluor Albus, tinct. iron in...... 650
Foster, Eugene ................ 219
Fractures, management of ...... 415
Frontier Doctor................. 571
Gall Stones.................... 569
Gelseminum.................... 570
  In cough .................. 757
Genital irritation............... 623
Genu-Pectural Posture, in uterine disease ...................... 449
Geology and Bible.......... .... 124
German Hospital, diseases of ... 343
Gleanings. ................... 695
Gonorrhœa, cutting short of.... 168
  In infants................. 170
Gotham, Wise Men of.......... 306
Greene, W. A.................. 534
Greeting....................... 634
Hæmorrhage, arterial, tincture of iron in ...................... 21
  Nasal ..................... 411
  Uterine.................... 45
Hair Restorative................ 400
Hammocks for Invalids......... 246
Headache, different forms of.... 415
  Functional................. 561
Hæmaturia, malarial............ 539
Hemi-chorea .... .............. 658
Hemorrhoids, case of........... 98
  Discussion of.............. 277
Hernia ........................ 65
  Strangulated, enema in..... 95
  New treatment of...... 310, 657
  Traumatic ventral.......... 502
Herpes......... .............. 276
Hints on Obstetric Practice...... 633
Homœopathy.. ................ 574
Honest Confession.............. 316
Honors Accumulating ......... 256
Hopkins, T. S............. 321, 449
Hospital Construction.......... 632
Hot Drinks.................... 415
Hoyt, W. D.................... 329
Human Physiology ............ 505
Hydrocele, treatment of......... 125
Hygiene, Report of U. S. A , 247, 375
Hysterical Seizures, treatment of 368, 566
Infantile Thumb-Sucking....... 426
Inferior Maxillary Bone, frac. of. 441
Inflammation, cure of .. ...... 526
International Medical Congress.. 638
Intestinal Obstruction.......... 94
  From malaria.............. 329
Intra-Rectal Examna'n, danger of 56
Iodo-Bromide Calcium, comp'n'd solution of.................. 367
  Elixir of.................. 287
Ipecachuanha, remarks on, 10, 80, 99, 136
  Vomiting pregnancy, for.... 28
  Intermittents........... 99, 200
  Dysentery ................ 270
Iridotomy ..................... 440
Jaborandi ..................... 568
Jaw, closure of................. 577
Jealousies of Profession ........ 702
Jelks, E. A..................... 462

Johnson, Jno. M., 168, 384, 650, 653, 702
Johnson, Jno. Thad............ 705

Kendrick, W. S. ............... 704
King Ferdinand,............... 214

Labor, anæsthesia in........... 386
Contraction in ....... .... 389
Horrible ...... ........... 694
Support in................. 197
Laborer Worthy of his hire. 245
Ladies and the Journal.......... 63
Left Kindey, gangrene of........ 425
Le Hardy, Henry............... 468
Lewis, M .................. 28
Lightning rods................. 309
Stroke .................... 374
Liquor, sennæ ................. 365
Lithotomy, cases of........ 193, 462
Lochia, failure of.............. 655
Logan, J. H.................... 1
Logan, J. P.................... 525
Lewis, R. M. .................. 464
Louisville Medical News........ 633
Lung, destruction of ........... 687

Mammitis...................... 646
Mania, acute, ergotine in........ 559
Mason, E...................... 389
May, M. R..................... 391
Mania a-potu.................. 460
M. D., use of.................. 61
Medical Association of Georgia, 115, 512, 127
Annual Meeting of.......... 764
Alabama ............... 109, 191
Mississippi, trans. of....... 759
Protection Association, New Orleans.................. 301
South Georgia......... 317, 511
Ohio ...................... 303
Medical Society, D. C., trans. of 507
Illinois, trans. of........... 632
Colorado, trans. of ......... 759
Association of Pennsylvania, trans. of................. 571
Medical Chirurg. Faculty, Md., trans. of................. 440
Medical Legislation. ........ 47, 365
Diagnosis.................. 698
Education and legislation... 299
Evidence........ ......... 568
Papers. burial of. .......... 49
Legend.................... 249
Medicine, Law and Theology.... 501
Medico-Surgical Evidence in New Born Child .................. 759
Menstruation, What is it? ...... 305
Physiology of.............. 318
Vicarious.................. 99
Meningitis, cerebro-spinal...... 168

Mercury, actions of......... 63, 104
Miasma, treatment of........... 568
Michigan Trouble.............. 410
Miscellaneous cases, reports of.. 368
Mistaken pregnancy. .......... 49
Minor Surgery and Bandaging .. 506
Modern Education ............. 371
Moore, K. P................... 386
Monstrosity.................... 625
Morphia and Atropia........... 99
Solution of................ 567
Myalgia, case of............... 273

Narrow, Escapes............... 79
Nervous System and Revulsives . 503
Lectures on diseases of ..... 758
Notes of................... 430
Neuralgia, gelsea in ............ 570
Newspaper Charlatan........... 237
New pocket case ........... ... 534

Obstetrics, anæsthetics in....... 225
Obstruction, intestinal.......... 269
Odontalgia ... ................ 123
Oleomargarine. ..... ......... 296
On Dit ........................ 635
Ophthalmia, symptoms, treatment of.. .............. 154, 156
Opium, antidote for, 99, 161, 653, 655
Poisoning......... 653, 661, 731
Intoxication .... .......... 245
Optic Nerve, treatment of....... 154
Otorrhœa, salicilic acid in....... 689
Styptic cotton in........... 756
Ovarian Cyst..............159, 164
Ovariotomy............153, 229, 232
Autopsy in ................ 29
Ovulation and Menstruation..... 713
Ovum, discharge of............. 500
Ozena, treatment of............ 567

Paraphymosis, reduction of ..... 272
Pat's Criticism ............... 186
Perineum, rupture of ...... 100, 161
Peritoneal Wounds............. 221
Pessary, new ................. 275
Pharmacy in Georgia.. ........ 314
Plegmasia Dolens.............. 76
Phymosal Paraplegia........... 399
Physicians and Druggists, relations of..................... 214
Visiting list................ 507
Pacenta, abortion in............ 48
Adherent ......... 74, 265, 692
Plaster, new................362, 412
Pneumatic Self-Repositor....... 230
Pneumonia, treatment of........ 52
Poisons, Taylor................ 631
Polar and Tropical Worlds...... 758
Polypus, Uterine............... 222
Does it return.............. 271
Nasal...................... 297

Pooley, J. H. .................... 65
Popular Health Almanac......... 295
Position, Pneumatic Pressure... 129
Post-Partum Pills............. 627
Potash Silicate............... 695
Practice of Medicine, Cyclopœdia of.............. 311, 631, 758
Prayer as Medicine............. 371
Pregnancy, Vomiting of, treatment.........28, 302, 65, 617, 688
Mistaken .................. 49
Albuminuria in ............ 616
Private Practice, Notes from.... 335
Prize Essay..................... 191
Phthisis, Cough of.............. 46
Effusions in ............... 566
Pulmonary Diseases, Dry Air in. 262
Puerperal Women.............. 102
Purpura Hemorrhagia, ergot in . 748

Quartet.................. 51
Quacks and Diplomas........292, 576
Doctor ........................ 369

Rauschenberg, Ch............... 713
Render unto Cæsar............. 311
Retina, Detachment of ......... 157
Rheumatism, treatment of ...... 405
New Method................ 628
Carbolic Acid in............ 629
Salicylic acid in........... 757
Rheumatic Fever, treatment of.. 691
Rives, B. R.................... 265
Rolling Stone.................. 572
Rupture, Intestines............. 438

Salacine in Diarrhœa........... 757
Savannah, Association at ....... 127
Science ........................ 3[illegible]2
Scrofulides, treatment of........ 759
Sims, J. Marion................ 296
Skin Grafting, case of .......... 641
Sleeplessness ................. 569
Somnambulist .................. 286
Sores, Venereal, treatment of.... 423
Spaying Sows.................. 221
Special Committee, report of.... 513
Spine, Curvature of............. 405
Spleen, Enlarged............... 55
Sponge Tents.................. 239
Squibb's Ether.................. 272
Starr, L. E..................... 152
State Societies ................. 63
State Board Health, 250, 255, 381, 384, 513, 700, 760
Strangury ...................... 152
Summer Complaint............. 436
Suppurating Tumors............ 58
Syphilitic Infection............. 691
Syphilis, Lecturers on.......... 506

Tape Worm, removal of ........ 626
Tetanus......................... 430
The Physicians of Oldham county, Ky......................... 508
The Body and its Aliments...... 699
Thomas, J. G................... 335
Tincture Iron................... 271
Tonsillar Hypertrophy.......... 610
Tonsils and Uvula, excision of .. 688
Toughened Glass.............. 373
Trichinosis, Sutton on.......... 366
Triplets, case of................ 694
Tumor, Cystic .................. 164
Lobulated.................. 656
Twins, what of................. 401

Uræmic Poisoning ............. 604
Urethra, Stricture of............ 622
Urethritis, Acute................ 657
Treatment of............... 705
Uterine Conception, Quinine in.. 182
Polypus .................... 222
Uterus, Double ................. 662

Vaccination.................... 693
Vaginismus..................... 692
Varicella and Variola, connection between....................... 29
Viburnum ...................... 538
Vision, Optical Defects.......... 507

Warm Springs...............378, 441
Washington, B. H. ...........76, 531
Westmoreland, J. G..........63, 590
Westmoreland, W. F....577, 596, 673
Wetherly, J. S.................. 182
What's in a Name .. .......... 304
Woodhull, A. A......10, 80, 136, 200

Zinc, Phosphide of............. 564

